# Atypical HUS and Crohn’s disease—interference of intestinal disease activity with complement-blocking treatment

**DOI:** 10.1007/s00467-021-05167-9

**Published:** 2021-07-30

**Authors:** Orsolya Horváth, Kata Kelen, Zoltán Prohászka, Ádám Hosszú, Attila J Szabó, George S Reusz

**Affiliations:** 1 First Department of Pediatrics, Semmelweis University, HU 1083 Budapest, Hungary; 2 Pediatric Hematology and Stem Cell Transplantation Unit, Central Hospital of Southern Pest National Institute of Hematology and Infectious Diseases, Budapest, Hungary; 3 Research Laboratory, Department of Internal Medicine and Hematology, Semmelweis University, Budapest, Hungary

**Keywords:** Hemolytic-uremic syndrome (HUS), Thrombotic microangiopathy (TMA), Inflammatory bowel disease (IBD), Crohn’s disease (CD), Treatment

## Abstract

**Background:**

In atypical hemolytic-uremic syndrome (aHUS), various defects of the complement system have been reported to explain pathophysiology. Therapeutic options for complement inhibition are well-recognized; however, the links between various immune-derived diseases and aHUS are unclear, and their interference with treatment efficacy during long-term complement-blocking therapy is scarcely known.

**Case-diagnosis/treatment:**

We present a pediatric patient who developed aHUS with acute kidney injury in parallel with the onset of Crohn’s disease (CD), and who required long-term complement-blocking therapy with eculizumab (ECU). Unexpectedly, during the 6-year ECU treatment, an important intra-patient variation of the degree of complement inhibition was observed. In spite of continuous and stable doses of complement-blocking therapy, periods of incomplete blockade were observed in strong association with relapses of CD. When conventional and later biological therapy with adalimumab was introduced, with CD going into remission, complement blockade became complete again. Despite periodically low ECU levels and insufficient complement inhibition, no clinical or hematological signs of aHUS recurrence were detected during CD relapses.

**Conclusion:**

In aHUS cases secondary to CD, close monitoring of both complement inhibition and serum ECU levels is needed as intestinal disease can interfere with complement-blocking treatment. Increased doses of ECU may be necessary to maintain therapeutic blood levels of ECU and full complement blockade, especially if the intestinal disease is not under control.

## Introduction

Atypical hemolytic-uremic syndrome (aHUS) is a severe condition. Early and appropriate diagnosis and treatment are needed to avoid chronic kidney injury [1]. Patients with a genetic susceptibility to aHUS are prone to thrombotic microangiopathy (TMA) recurrences and may require lifelong complement inhibition therapy [2]. Although various defects in the complement system explaining pathophysiology have been described, and therapeutic options for complement inhibition are well-recognized [3–6], the links between various immune-derived diseases and aHUS are unclear. We report a pediatric case in which the patient developed TMA during the first manifestations of Crohn’s disease (CD).

## Case report

### The patient’s story

Here we report the case of a girl followed between the ages of 13 and 18 years with CD and aHUS. The patient was born after an uneventful twin pregnancy. Her twin brother has severe CD with complications necessitating repeated surgery for abdominal abscesses and fistulas. The first symptoms in our patient started in 2014 at the age of 13 with recurrent diarrhea, mucus in the stool, weight loss, and anemia. Initial laboratory data suggested CD (no pathogens in the stool culture, stool calprotectin, and anti-Saccharomyces cerevisiae antibodies (ASCA) IgG were elevated (> 900 microgram/g and 149 U/L, respectively)). The parents did not agree to performing an endoscopy at this stage.

In parallel, the patient had anemia, normal thrombocytes, proteinuria—reaching nephrotic range—and hematuria, while serum creatinine started to increase rapidly (223 micromole/L). Abdominal ultrasound showed enlarged hyperechogenic kidneys. Kidney biopsy was performed. Two days after the kidney biopsy, the complete clinical picture of aHUS developed with thrombocytopenia, hemolytic anemia, low haptoglobin levels, acute kidney impairment (serum creatinine 263 micromole/L), and high lactate dehydrogenase levels. Indeed, kidney histology confirmed the presence of thrombotic microangiopathy.

After Enterohemorrhagic E. coli (EHEC)–induced HUS diagnosis was ruled out, complement and genetic diagnostics were performed to establish the etiology of aHUS. Treatment of TMA was urgent due to rapidly decreasing kidney function (estimated glomerular filtration rate 23 mL/min/m^2^) and low thrombocyte levels before any knowledge of the patient’s genetic background. Transfusions of fresh frozen plasma (FFP, 18 times), therapeutic plasma exchange sessions (4 times), and intravenous steroid shots (250 mg each, 3 times) were performed to eliminate eventual anti-factor H antibodies.

Hemolysis was stopped before the initial treatment, and hematological response was reached; however, proteinuria, hematuria persisted, and serum creatinine remained high. To avoid chronic kidney failure, complement-blocking therapy was urgent. Six weeks after the diagnosis of aHUS, complement blockade with eculizumab (ECU) became available and was started with an initial dose of 900 mg (for her weight of 38 kg).

Complement measurements (all pathway activities, complement activation products, and ADAMTS13 (a distintegrin and metalloprotease with thrombospondin-1-like domains, member 13)) were performed. Total classical (CH50, sheep red blood cell hemolytic titration, reference range: 48–103 CH50/mL) and alternative pathway (ALT, Wieslab Comp AP330 kit, 70-125%, Euro Diagnostica, Malmö, Sweden) activities and free serum ECU (in-house enzyme-linked immunosorbent assay from 2016 onward) levels were performed systematically directly before starting ECU infusion every 2 weeks. Fully suppressed CH50 and ALT activities were defined as an activity below 10%. Serum ECU concentrations of 50–100 microgram/mL were considered to achieve complete complement blockade [7].

Signs of effective complement blockade were detected without delay. Classical and alternative pathway activities were depleted. Low C3 and C4 levels, as signs of severe complement consumption, normalized (to 1.1 mg/L and 0.56 mg/L, respectively). Lower ADAMTS13 activity values (54–77%), as a sign of secondary endothelial damage [8], returned to normal (99–111%). ECU treatment was continued and a satisfying nephrological response was reached; creatinine returned to normal, and proteinuria decreased gradually to the normal range. Other, supportive aHUS therapies (PE, FFP) were stopped.

A thorough genetic study of the complement system was performed and revealed the presence of a previously undescribed “likely pathogenic” factor I variant (*R339L*). In silico prediction of variant pathogenicity was assessed, showing that the serine protease domain of human complement factor I was affected. In addition, CFH tgtgt and MCP ggaac aHUS risk haplotypes were detected. The twin brother carries the same factor I variant (*R339L*), the CFH tgtgt and MCP ggaac risk haplotypes. The mother bears the same CFI mutation and the MCP ggaac risk haplotype and is heterozygous for the CFH tgtgt risk haplotype. Family history was negative for aHUS despite this genetic background.

### Emerging problems

ECU therapy was started in 2014 and was continued systematically every 2 weeks during the entire study period. Maintenance ECU was initially 900 mg (every 2 weeks). Unexpectedly, a remarkable intra-patient variation of complement inhibition was observed. In spite of continuous complement-blocking therapy, peaks of complement pathway activities (CH50, ALT) were observed, the blockade was incomplete, and a strong association with the recurrence of bowel symptoms was found. In response, first the ECU dose was increased (to 1200 mg every 2 weeks, as the patient’s weight meanwhile had increased to 42 kg) assuming that the patient needed a higher dose due to weight gain. However, complement blockade was reduced even at higher doses during CD relapse. An ileum biopsy performed in April 2016 showed the typical histological picture of CD. Conventional treatment for CD (steroids, local steroids, and azathioprine) was started, with a rapid remission of CD. Non-compliance with conventional therapy resulted in CD recurrence in October 2017, coupled with the development of a perianal fistula. Biological treatment with adalimumab, a fully human, high-affinity, recombinant anti-tumor necrosis factor alpha monoclonal antibody [9], was introduced, which resulted in the remission of CD.

**Fig. 1:**
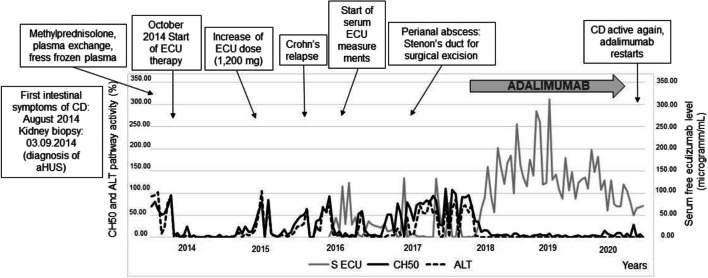
Complement classical and alternative pathway activities (from 2014 to 2020) and free eculizumab levels (from 2016 to 2020) (measured on day 14 after ECU infusion) during atypical hemolytic-uremic syndrome and Crohn’s disease treatment. Complete complement blockade is observed only during eculizumab and adalimumab therapy. Legend: aHUS, atypical hemolytic-uremic syndrome; ALT, alternative pathway activity; CH50, classical pathway activity; CD, Crohn’s disease; ECU, eculizumab; S ECU, serum free eculizumab level

Figure 1 shows the variation of complement activity with time (on day 14 after administering the prescribed dose and before the next dose) as a function of the course of intestinal disease. While CD was in remission, complete complement blockade was achieved, and CH50 and ALT pathway activities were below 10%. However, during CD recurrence, the efficacy of complement blockade decreased dramatically.

To determine the cause of this intra-patient variability, other potential factors were analyzed. Proteinuria had not been detected since 2015, and intestinal bleeding was not significant. Free ECU serum levels were analyzed shortly before the next ECU dose from 2016 onward. Serum levels decreased to subtherapeutic concentrations (< 8–10 microgram/mL) despite regular dosing. As shown in Fig. 1, with stable doses of ECU (1200 mg every 2 weeks), therapeutic levels (> 50 microgram/mL) were reached shortly after the start of adalimumab therapy. Despite low ECU levels and incomplete complement inhibition, no clinical or hematological signs of TMA activity were observed during the recurrence of CD. The patient’s weight was stable (between 50 and 55 kg) from the age of 16. The patient remained in hematologic and renal remission throughout the study period. C3 (median (1.2 mg/L (IQR 0.99–1.37)), C4 (0.4 mg/L (IQR 0.28–0.45)), and ADAMTS13 activity (median 101% (IQR 91–115%)) values were within the normal range from the start of ECU therapy.

## Discussion

This is a case of a pediatric patient who developed aHUS with acute kidney injury during an episode of CD, requiring long-term complement-blocking therapy. During long-term ECU treatment, a considerable intra-patient variability in the degree of complement blockade was observed along with incidents of CD. The ECU doses were adjusted for weight as recommended, but were too low for the patient with an ongoing inflammatory intestinal disease. Relapses and remissions induced characteristic fluctuations in the efficacy of complement blockade, with apparent ECU consumption during CD incidents. The temporary decrease in the effectiveness of the blockade was not associated with the return of the aHUS.

ECU is a humanized monoclonal antibody against complement C5 [1, 4]. A number of factors have been identified that can affect ECU clearance during long-term therapy, including substantial change in body weight, variable serum C5 levels during infections, or loss by proteinuria [10, 11]. In this case, the reason for decreased ECU activity is not clear. Possible explanations include local inflammation in the gut or intestinal ECU loss.

In addition, our patient had the same genetic background as her twin brother, both patients had CD, but only the girl developed aHUS. How far the likely pathogenic CFI variant had a role in the occurrence of aHUS in our patient with CD and in the remission of aHUS under ECU is uncertain.

To our knowledge, five aHUS cases associated with inflammatory bowel disease have been published in the literature [12–15]. Importantly, CD and colitis ulcerosa cases were also described with aHUS cases [12–15]. All five cases were treated with ECU successfully. To our knowledge, however, simultaneous serum-free ECU levels, CH50 and ALT activities, are described for the first time in our case. In two published cases considered secondary TMA, an attempt was made to stop ECU therapy. One patient had a simultaneous recurrence of aHUS and CD after discontinuation of ECU [12], and UC relapsed in another case after stopping ECU [14]. The pathomechanism of inflammatory bowel disease may hypothetically be analogous with stem cell transplantation–associated TMA with acute graft-versus-host disease. Higher and frequent doses of ECU are needed to resolve this secondary form of TMA; patients with intestinal involvement have the fastest ECU clearance [16]. Further studies are needed to clarify the mechanism of ECU consumption in the gut and the role of concurrent immune-mediated diseases during long-term complement-blocking therapy.

In conclusion, Crohn’s disease can be one of the possible causes of secondary aHUS in children. In aHUS cases secondary to CD, close monitoring of both complement inhibition and serum ECU levels is needed as intestinal disease can interfere with complement-blocking treatment. Increased doses of ECU may be necessary to maintain therapeutic blood levels of ECU and full complement blockade, especially if the intestinal disease is not under control.
